# Current clinical status of perioperative comfort therapy in pediatric anesthesia in China: a national cross-sectional survey

**DOI:** 10.3389/fped.2026.1843447

**Published:** 2026-06-22

**Authors:** Jing Shen, Lufeng Yang, Wenli Hou, Xiang Li, Qianyi Qiu, Fang Chen

**Affiliations:** 1 Department of Anesthesiology, Shenzhen Children's Hospital, Shenzhen, Guangdong, China; 2 Department of Anesthesiology, Shenzhen Children's Hospital, Southern University of Science and Technology, Shenzhen, China; 3 Department of Clinical Medicine, Medical School, Shenzhen University, Shenzhen, Guangdong, China

**Keywords:** emergence delirium, parental presence, pediatric anesthesia, perioperative comfort therapy, preoperative anxiety

## Abstract

**Objective:**

To investigate the current implementation of and key barriers to perioperative comfort therapy in pediatric anesthesia in China, and to provide evidence-based support for optimizing the perioperative anesthesia experience of children.

**Methods:**

A cross-sectional online survey was conducted from March 21 to May 21, 2025. Using convenience sampling, an anonymous electronic questionnaire was distributed to anesthesiologists nationwide via the WeChat group of the Pediatric Anesthesia Branch of the Chinese Society of Cardiothoracic and Vascular Anesthesia. Participants were invited to complete the survey anonymously and were requested to provide objective institutional data as completely and accurately as possible. The questionnaire comprised 22 closed-ended questions and 3 open-ended questions, covering preoperative and postoperative anesthesia management, parental presence rates, and other relevant indicators. Data analysis and visualization were performed using SPSS version 27.0 and GraphPad Prism 10.

**Results:**

A total of 150 valid responses were collected. Participating hospitals were predominantly tertiary A-grade facilities, accounting for 79.3% of the sample. Preoperative visits were universally implemented (100%) and preoperative education, fasting instructions, and explanations of anesthesia plans were provided in 95.3%, 94.0%, and 92.7% of hospitals, respectively, non-routine premedication remained the most common anxiolytic strategy, reported by 59.3% of hospitals. When premedication was used, dexmedetomidine, midazolam, and ketamine or esketamine were the main agents, reported by 29.3%, 25.3%, and 20.0% of hospitals, respectively. Non-pharmacological interventions were widely adopted, including comfort toys or picture books in 84.4%, play therapy in 44.2%, and educational videos in 36.1% of hospitals, whereas virtual reality was rarely used, with a utilization rate of 9.5%. Parental presence was permitted during anesthesia induction in 30.0% of hospitals and in the post-anesthesia care unit (PACU) in 20.7%. Only 12.7% of hospitals had dedicated induction rooms. The main barriers to parental presence were concerns regarding parental emotional distress, reported by 75.3% of respondents, and increased workload, reported by 73.3%. Notably, only 34.0% of hospitals used standardized scales to assess emergence delirium. Propofol was the most commonly used intervention for emergence delirium, accounting for 48.0%, while early parental comforting accounted for 21.3%.

**Conclusions:**

In China, current pediatric perioperative anesthesia management remains insufficient in addressing children's psychological well-being, and interventions aimed at reducing perioperative anxiety are suboptimal. These shortcomings are mainly reflected in three areas: first, inadequate assessment of children's anxiety during preoperative education and insufficient preoperative anxiolytic measures; second, a substantial discrepancy between recognition of the importance of parental presence during anesthesia induction and recovery and its limited implementation in clinical practice; and third, underuse of standardized assessment scales for emergence delirium, together with limited diversity in management strategies. These limitations are likely attributable to constraints in medical resources and insufficient staffing.

## 1 Background

Pediatric perioperative comfort therapy is a child-centered, multidimensional, and comprehensive perioperative medical concept and practice system (1, 2). It primarily focuses on the management of physiological pain, as well as the mitigation of psychological anxiety and fear. Its main objective is to provide children with a safe, stable, minimally painful, and ideally fear-free perioperative experience (3–5), thereby maximizing protection of their physical and mental health.

Unlike adults, children exhibit immature cognitive development and high parental dependency, rendering them particularly vulnerable to preoperative anxiety (6, 7). Children may also experience significant anxiety during anesthesia induction and are at high risk for common postoperative complications, such as emergence delirium (7, 8). These issues not only affect children's experience of medical therapy but also prolong postoperative recovery and may even have adverse effects on their long-term psychological well-being in relation to medical treatment (4, 9, 10). As contemporary concepts in pediatric anesthesia continue to evolve, anesthesiologists are paying increasing attention to patient comfort and psychological interventions for children (11, 12).

By the end of 2023, there were approximately 260 million children in China (13), with millions undergoing surgical procedures annually (14–16). Given this large volume of pediatric surgical procedures, standardized and streamlined management of perioperative comfort therapy for children has become increasingly urgent. Several effective assessment systems are currently available, including the Modified Yale Preoperative Anxiety Scale (m-YPAS) (17), the Visual Analogue Scale (VAS) (18), structured assessment tools for postoperative delirium such as the Pediatric Anesthesia Emergence Delirium (PAED) scale (19), and the Emergence Agitation Risk Scale (EARS) (20), which have been considered as the standardized core basis for the precise screening and assessment of perioperative anxiety in children (21). Recent psychometric evaluations have confirmed their favorable validity and reliability (22, 23). Furthermore, parental presence during anesthesia induction and emergence recovery has been shown to effectively reduce perioperative psychological stress in children (24, 25).

Despite the robust evidence base and well-established clinical value of comfort-oriented measures, standardized pediatric perioperative comfort therapy has not yet been fully realized in China (26, 27), largely owing to the uneven distribution of medical resources across the country. Although several domestic investigations have been conducted, most consist of studies are single-center controlled trials or observational studies that primarily evaluate the efficacy of isolated interventions (28, 29). Crucially, there is a paucity of multicenter national data characterizing current clinical practices among anesthesiologists in pediatric institutions. To address this gap, this study aimed to delineate the current landscape of perioperative comfort care in Chinese pediatric anesthesia, specifically examining preoperative anxiety management, parental presence, and emergence delirium management, thereby providing evidence-based guidance for optimizing perioperative therapy.

## 2 Materials and methods

### 2.1 Study design

We conducted a national cross-sectional online survey on pediatric anesthesia practices in China. This study is reported in accordance with the Checklist for Reporting Results of Internet E-Surveys (CHERRIES) guidelines (30). Ethical approval was granted by the Ethics Committee of Shenzhen Children's Hospital, Shenzhen, China, with approval number 202502501.

The survey introduction explicitly stated that participation was voluntary and that completion of the survey implied informed consent. The cover letter clearly indicated that no personal names would be collected, that there were no right or wrong answers, and that responses would not be linked to performance evaluations. Questionnaire items were worded neutrally, and projective questioning was applied to sensitive topics. The specific content of the questionnaire was accessible only to the study investigators, and participants could not view responses from others. Data were kept confidential, and participants could withdraw at any time without inducement or coercion. In addition, automatic de-identification, with no collection of IP addresses or names, ensured anonymity.

Participating physicians were required to meet the following inclusion criteria: (1) being licensed anesthesiologists engaged in pediatric anesthesia; (2) voluntarily participating in this survey; and (3) being affiliated with a hospital that has a pediatric anesthesia specialty.

### 2.2 Questionnaire development and validation

The questionnaire was developed by three senior pediatric anesthesiologists. Through logical analysis, all team members jointly refined the skip logic and relational logic and conducted consistency checks to ensure data quality and standardize item phrasing. The questionnaire consisted primarily of 22 closed-ended questions, including single-choice and multiple-choice items, and 3 open-ended questions. The questionnaire covered the following areas: (1) baseline information of the respondents; (2) preoperative anesthesia management measures, including models and content of preoperative education and interventions for relieving preoperative anxiety in children; (3) postoperative anesthesia management measures, including the use of emergence delirium assessment scales, management strategies, and whether parental presence was permitted; and (4) limiting factors affecting the implementation of comfort therapy in Chinese hospitals. The complete questionnaire is provided in Supplementary Material 1.

Regarding mandatory and optional settings, all closed-ended questions were set as required items to ensure data completeness, avoid missing key information, and guarantee the quality of the sample data. Only three open-ended supplementary questions were set as optional items, allowing respondents to provide additional suggestions based on their clinical experience. These questions served only as supplementary material and were not included in the statistical analysis, thereby maintaining a balance between questionnaire completeness and response flexibility.

Regarding questionnaire skip logic, reasonable logical branching and conditional screening rules were established. Targeted skip logic was applied to certain subgroup questions, automatically hiding irrelevant items to prevent respondents from answering unrelated questions. In addition, logic consistency checks were implemented throughout the questionnaire to avoid contradictory responses or random selections.

### 2.3 Pilot study

Before nationwide rollout, a two-phase pilot study was conducted.

#### 2.3.1 Phase 1: cognitive interviews

From February 15 to February 28, 2025, cognitive interviews were conducted with 10 anesthesiologists from three hospitals, including 5 senior and 5 junior anesthesiologists. The interviews assessed question comprehension, clarity of terminology, and response burden.

#### 2.3.2 Phase 2: technical pilot test

From March 1 to March 10, 2025, a technical pilot test was conducted with 30 anesthesiologists. The questionnaire link was sent via the researcher's personal WeChat account, accompanied by a brief explanation of the study purpose, voluntary participation, and anonymous completion. An open-ended question was added at the end of the questionnaire: “Which questions do you find unclear, have incomplete options, or are difficult to answer? Please specify the question number and your suggestions.”

### 2.4 Sampling and recruitment

Convenience sampling was used through the WeChat group of the Pediatric Anesthesia Branch of the Chinese Society of Cardiothoracic and Vascular Anesthesia. The cover letter of the questionnaire explicitly informed participants that participation was voluntary, anonymity was guaranteed, and no personally identifiable information, such as name, employee ID, or telephone number, would be collected. No incentives were provided, and submission of the completed questionnaire implied informed consent. All participants were requested to provide objective statistical data from their respective institutions as completely and accurately as possible.

### 2.5 Data collection and management

The questionnaire was officially launched on March 21, 2025, and data collection was completed on May 21, 2025. Responses that were incomplete, had excessively short or long completion times, contained logical contradictions, or showed obvious random answering patterns were excluded. When two or more responses were submitted from the same hospital, only the questionnaire from the respondent with longer clinical work experience was retained. Respondents with longer work experience and richer clinical expertise were considered to have a more accurate understanding of their hospital's actual clinical situation.

Before analysis, the collected dataset underwent rigorous cleaning. Missing data were minimal, with an item non-response rate of less than 2%, and were addressed using listwise deletion in the respective analyses. For the open-ended questions, similar themes were merged, and frequencies were reported by category. Descriptive statistics were performed using SPSS version 27.0 (IBM Corp., Armonk, NY, USA) to calculate frequencies and percentages for each indicator. GraphPad Prism 10 (GraphPad Software, Boston, MA, USA) was used to generate figures for result presentation.

## 3 Results

### 3.1 Results of the phase 1 pilot test

Based on feedback from 10 anesthesiologists, the skip logic was simplified. For example, selecting “no parental presence” automatically bypassed subsequent questions related to parental presence. In addition, “select all that apply” options were added to allow multiple responses where appropriate.

### 3.2 Results of the phase 2 pilot test

In the second phase, 30 questionnaires were distributed and all 30 were returned, the questionnaire completion rate was 100%, after excluding 5 questionnaires with completion times that were either too long or too short, a total of 25 valid responses were collected. Based on participant feedback, the following issues were identified and addressed: (1) basic information items on hospital level were added; (2) response options for certain questions were added based on clinical practice, such as the use of new drugs and emerging technologies; (3) questions were reordered to improve logical flow; and (4) quality control criteria were established. Responses with completion times of less than 4 min or more than 15 min were excluded, and open-ended answers with fewer than 10 characters and lacking meaningful content were considered invalid.

### 3.3 Results of the formal questionnaire survey

A total of 400 invitations were distributed through the society's channels, and 200 responses were received, corresponding to a response rate of 50.0%. After applying the exclusion criteria, 150 valid questionnaires were finally obtained. The detailed data are as follows.

#### 3.3.1 Basic information analysis

A total of 150 hospitals participated in the questionnaire survey. Most participating hospitals were tertiary A-grade hospitals, accounting for 79.3% of the sample. Among respondents, 80.0% held senior professional titles, including chief physician and associate chief physician. Regarding clinical experience, 82.7% had practiced for more than 10 years, 12.0% for 6–10 years, 3.3% for 3–5 years, and 2.0% for fewer than 3 years. Detailed information is shown in Table 1.

**Table 1:** Demographic and professional characteristics of survey participants and their hospital level (*N* = 150).

Characteristics	Categories	*n*	Percentage (%)
Regional Distribution	East China	52	34.7
	Central China	28	18.7
	South China	25	16.7
	Southwest China	18	12.0
	North China	15	10.0
	Northeast China	12	8.0
Professional Title	Chief Physician	63	42.0
	Associate Chief Physician	57	38.0
	Attending Physician	24	16.0
	Resident Physician	6	4.0
Working Experience	>10 years	124	82.7
	6–10 years	18	12.0
	3–5 years	5	3.3
	<3 years	3	2.0
Hospital Level	Class III Grade A	119	79.3
	Class III Grade B	13	8.7
	Class II Hospital	13	8.7
	Others	4	2.7

#### 3.3.2 Preoperative anxiety assessment and management

##### 3.3.2.1 Preoperative anxiety assessment

All participating hospitals (100%) conducted preoperative visits for elective surgery, and 95.3% provided detailed preoperative education. The main educational content included explanations of the anesthesia plan (92.7%) and fasting instructions (94.0%). In contrast, parental education and anxiety assessment received less attention, with implementation rates of 28.0% and 19.3%, respectively.

##### 3.3.2.2 Premedication and non-pharmacological interventions

Non-routine premedication remained the most common anxiolytic strategy, reported by 59.3% of hospitals. When premedication was used, the most commonly used premedications were dexmedetomidine (29.3%), midazolam (25.3%), and ketamine or esketamine (20.0%). Notably, remimazolam was used in only one institution as an alternative to conventional sedatives.

**Figure 1:**
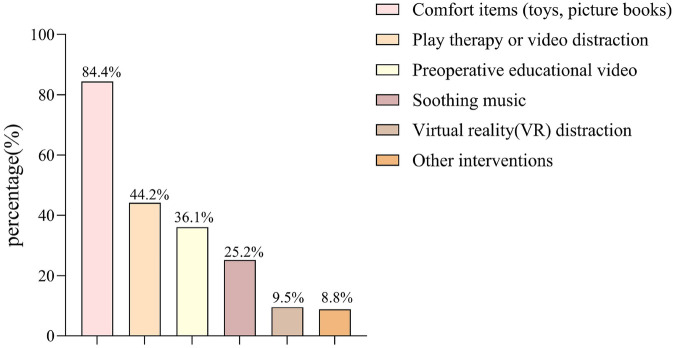
Distribution of non-pharmacological interventions used for pediatric preoperative anxiety.

**Figure 2:**
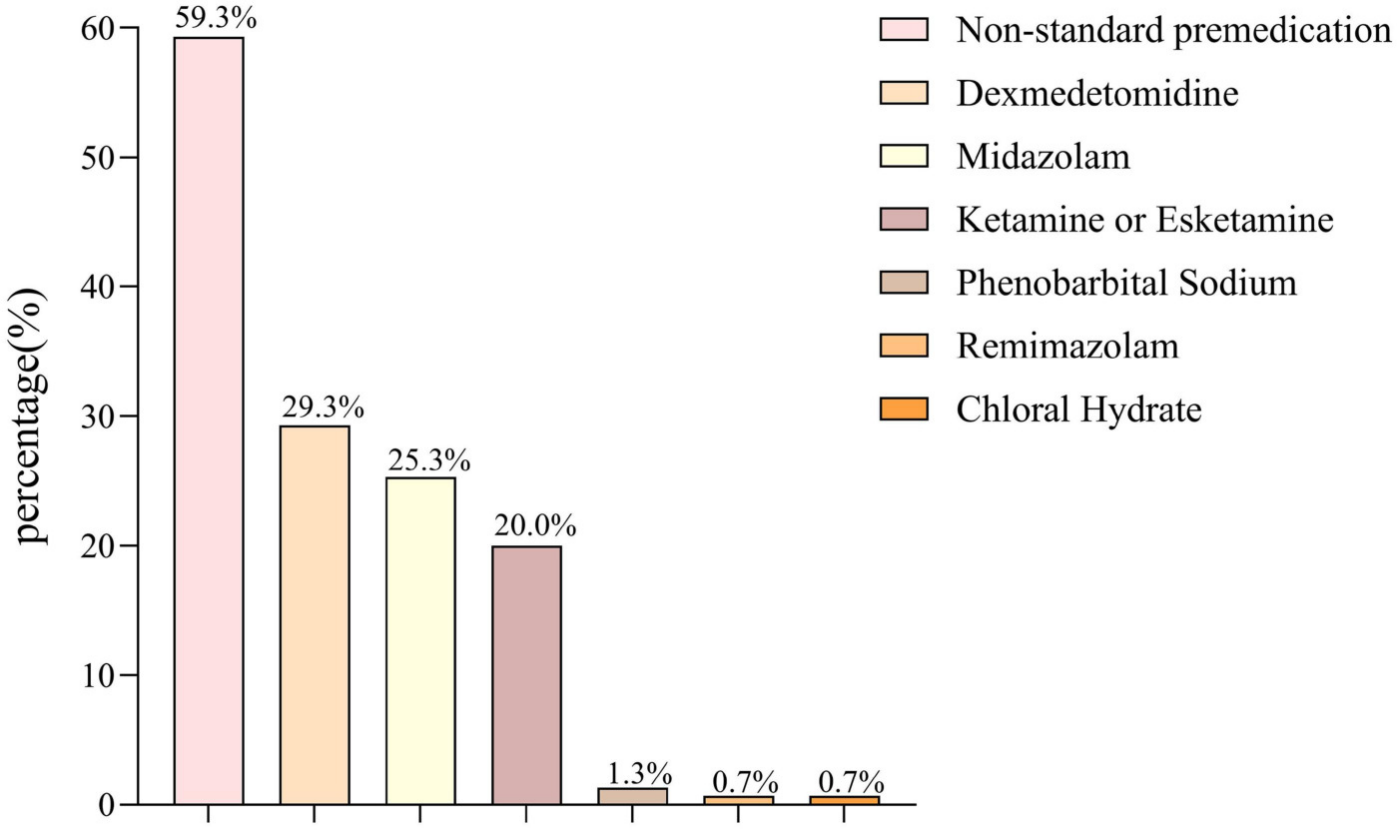
Distribution of pharmacological premedication for pediatric preoperative anxiety.

Among non-pharmacological interventions, comfort toys or picture books were the most widely used (84.4%), followed by play therapy or video distraction (44.2%). In contrast, virtual reality (VR) was used in only 9.5% of cases. Details of the premedication and non–pharmacological interventions are presented in Figures 1, 2.

##### 3.3.2.3 Anesthesia induction

Propofol (90.0%), midazolam (49.3%), and ketamine or esketamine (44.7%) were the main drugs used for anesthesia induction. By contrast, dedicated induction rooms were available in only 12.7% of hospitals.

#### 3.3.3 Postoperative delirium management and assessment

##### 3.3.3.1 Assessment of emergence delirium

Only 34.0% of hospitals used the PAED scale to assess postoperative emergence delirium. The incidence of emergence delirium was reported as low risk (<10%) in 58.0% of hospitals and as moderate-to-high risk (>=10%) in 22.7%. The remaining 19.3% of hospitals did not perform any assessment.

##### 3.3.3.2 Management of emergence delirium

Propofol was the most commonly used intervention for the management of emergence delirium (48.0%). This was followed by early parental comforting (21.3%), additional analgesics (12.7%), dexmedetomidine (12.0%), and physical restraint (3.3%).

#### 3.3.4 Parental presence

##### 3.3.4.1 Parental presence at induction

Parental presence was permitted during anesthesia induction in 30.0% of hospitals. Concerns about parental emotional distress (75.3%) and increased pressure on medical staff (73.3%) were the two most common barriers, outweighing traditional considerations such as workflow disruption (52.7%) and increased infection risk (44.0%).

##### 3.3.4.2 Parental presence in the post-anesthesia care unit

During the anesthetic recovery phase, 20.7% of hospitals allowed parental presence in the post-anesthesia care unit. However, most hospitals imposed conditional restrictions, such as limiting parental presence to younger children or those with emergence delirium (13.3%), or requiring parental training (38.0%).

**Figure 3:**
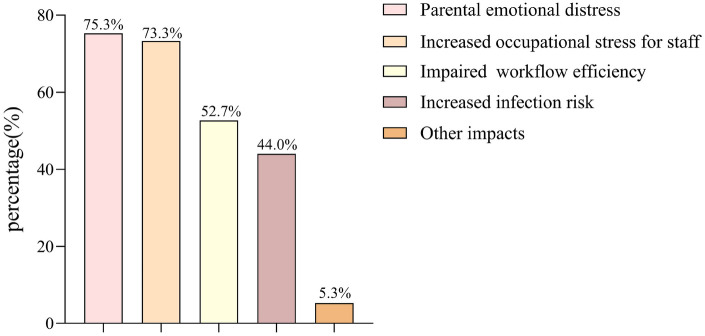
Medical staff’ concerns regarding parental presence during anesthesia induction.

**Figure 4:**
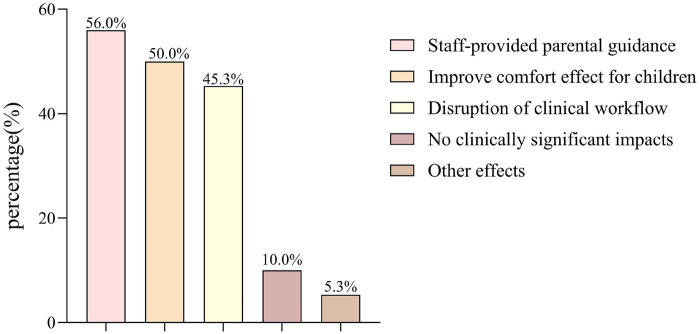
Perceived impacts of parental presence on clinical work among medical staff.

Healthcare professionals’ perceptions of the impact of parental presence during recovery were twofold. More than half of the hospitals (56.0%) believed that parental presence would require dedicated staff to guide parents. At the same time, 50.0% of hospitals acknowledged that parental presence significantly enhanced the calming effect on children. In addition, 45.3% of hospitals were concerned that it might disrupt clinical order. Medical staff's perceptions of the impact of parental presence on clinical workflow are shown in Figures 3, 4. And details regarding the implementation of parental presence and emergence delirium control measures are shown in Table 2.

**Table 2:** Implementation of parental presence and postoperative delirium control measures (*N* = 150).

Variable	Category	*n*	Percentage (%)
Parental presence at anesthesia induction	Permitted	45	30.0
	Not permitted	105	70.0
Dedicated induction room availability	Present	19	12.7
	Absent	131	87.3
Parental presence in PACU	Permitted	31	20.7
	Not permitted	119	79.3
Early parental comforting implemented for emergence agitation	Present	32	21.3
	Absent	118	78.7
PAED scale use for agitation assessment	Applied	51	34.0
	Not applied	99	66.0

PACU, post-anesthesia care unit; PAED, Pediatric Anesthesia Emergence Delirium.

#### 3.3.5 Factors impeding the implementation of comfort therapy

A shortage of qualified staff was the most frequently reported challenge, accounting for 41.3%. This was followed by limited pharmacological options (18.0%) and insufficient patient and parental education (16.0%). Other reported barriers included suboptimal environmental and facility conditions (12.7%) and a lack of standardized assessment tools (10.7%).

## 4 Discussion

This study reveals the current status of perioperative comfort therapy in pediatric anesthesia in China. Preoperative education and preoperative visits are generally well established; however, deficiencies remain in the assessment and management of pediatric anxiety and emergence delirium, parental presence, and the use of emerging technologies.

The results show that, in China, basic preoperative visits and safety education for pediatric anesthesia are generally well established, but attention to the psychological needs of children remains insufficient. Preoperative visits were completed one day before surgery in 98.0% of hospitals, with the remaining 2.0% completing them on the day of surgery. The preoperative education rate was 95.3%, with education mainly focusing on fasting and fluid restriction times (94.0% of hospitals) and anesthesia plans (92.7%). These findings indicate that a relatively comprehensive preoperative visit process has been established. However, parental education and preoperative anxiety assessment received less attention, with implementation rates of only 28.0% and 19.3%, respectively.

Currently, the international community places increasing emphasis on pediatric preoperative anxiety, and several national anesthesiology societies have included preoperative anxiety assessment as an important indicator of pediatric anesthesia quality (31, 32). For example, the m-YPAS is used to assess anxiety levels in children of different ages and to develop individualized intervention strategies accordingly (33, 34). In contrast, Chinese hospitals do not routinely conduct preoperative anxiety assessments for pediatric patients (35, 36). This disparity underscores the need to incorporate anxiety screening tools into routine clinical practice in China.

Furthermore, studies indicate that only 18.0% of hospitals in China provide preoperative education on risk factors for emergence delirium, while 34.0% use the PAED scale to assess emergence delirium; nearly one-fifth (19.3%) of hospitals do not conduct systematic assessments of emergence delirium at all. Although the majority of hospitals (58.0%) reported low (<10%) incidence rates of delirium, the widespread lack of assessment may obscure the true scope of the problem, and emergence delirium in pediatric patients has yet to become a routine indicator for monitoring healthcare quality.

The PAED scale has been developed and validated as a reliable tool for the assessment of emergence delirium. Recent psychometric evaluation has further confirmed its diagnostic accuracy [area under the curve (AUC) = 0.89] and inter-rater reliability (37, 38). It can dynamically reflect improvements or deteriorations in the quality of emergence and provide a quantitative basis for clinical intervention. Relevant studies have noted that, despite the PAED scale being the gold standard for assessing emergence delirium, a significant gap exists between awareness and its use in clinical practice (39). Only about one-third of hospitals routinely using the PAED scale for assessment, and a standardized evaluation process has not yet been established.

The lack of attention to preoperative anxiety and the deficiencies in the assessment and management of emergence delirium reveal a gap in addressing the psychological needs of pediatric patients in current perioperative comfort therapy in China. Therefore, shifting from a “physiological safety first” model to one that equally emphasizes both physiological and psychological well-being is the direction in which improvements are needed.

First, anesthesia healthcare professionals need to strengthen their attention to children's psychological and emotional states and improve early recognition of pediatric anxiety. Additionally, standardized assessment tools for anxiety and emergence delirium should be widely adopted and integrated into routine preoperative visit protocols to identify high-risk children.

In terms of parental presence rates, only 30.0% of hospitals allowed parental presence during anesthesia induction, and 20.7% allowed parental presence in the PACU. The main reasons for the low parental presence rates were concerns about potential parental emotional distress (75.3%) and increased workload for staff (73.3%). These figures stand in contrast to the recent survey showing that most regions in Europe allow parental presence (40–42). Low parental presence not only undermines children's sense of security but also limits the effectiveness of non-pharmacological interventions. Successful implementation models in Europe indicate that well-designed parental presence preparation programs, including preoperative education and dedicated staff, can reduce parental emotional instability and workflow disruptions (43, 44).

The low parental presence rate in China may be attributed to the fact that only 12.7% of hospitals are equipped with dedicated induction rooms for parental presence, along with a shortage of specialized guiding personnel, which limits parental involvement. Nevertheless, we believe that parental presence is feasible in China, as half of the hospitals (50.0%) acknowledged its value, recognizing that it significantly enhances the calming effect on children. To increase perioperative parental presence rates, China could improve infrastructure by providing dedicated induction rooms and training specialized guiding personnel. In addition, preliminary simulations at tertiary A-grade hospitals can be conducted to develop efficient workflow templates for widespread implementation at primary therapy hospitals.

Additionally, among non-pharmacological interventions, comfort toys or picture books were the most widely used (84.4%), while newer technologies such as virtual reality were used in only 9.5% of hospitals. This low level of adoption contrasts with findings from recent network meta-analyses, which showed that virtual reality was more effective than two-dimensional videos in reducing preoperative anxiety and postoperative pain in pediatric patients (45–47). Neurophysiological studies have also shown that virtual reality may modulate pain pathways through attentional distraction and cognitive reappraisal (48). Nevertheless, this emerging technology has not yet been widely integrated into clinical practice.

In terms of pharmacological interventions, the preoperative medication strategy was dominated by “not routinely used” (59.3%), which contrasts with the routine premedication rate of approximately 80% reported in Western pediatric centers (49). However, for selected children (e.g., those with extreme anxiety), dexmedetomidine (29.3%) or midazolam (25.3%) could be considered as preferred options, while the use of newer agents, including remimazolam, remained very low (0.7%) (50).

Differences across hospital levels in equipment availability, budget constraints, workflow integration, and personnel training unique to the Chinese healthcare environment may limit the implementation of emerging technologies and drugs. For example, the use of virtual reality to alleviate preoperative anxiety is concentrated in tertiary A-grade hospitals, suggesting that resource feasibility may be a determining factor.

## 5 Limitations

This survey has several limitations. First, its cross-sectional design precludes causal inference, and the use of self-reported data may have introduced reporting bias; convenience sampling may also have led to selection bias. Second, the sample may not have been fully representative in terms of geographic and institutional distribution. Secondary hospitals were underrepresented (8.7%), as were hospitals from northwestern China (7.3%). This incomplete coverage may have contributed to the overrepresentation of tertiary A-grade hospitals (79.3% of the sample) and, consequently, to an underestimation of the challenges faced in resource-limited settings. Consequently, the generalizability of the findings to smaller or geographically remote hospitals remains uncertain.

In addition, the respondents were predominantly senior clinicians: 82.7% had more than 10 years of professional experience, and 80.0% held the titles of chief physician or associate chief physician. These proportions exceed the typical professional distribution among anesthesiologists in China. Although this may have strengthened the quality and depth of the responses, it also suggests that the shortcomings identified in this survey are likely to reflect broader challenges in pediatric anesthesia practice in China, rather than merely deficiencies in the capabilities of junior or primary-level physicians.

Furthermore, since frontline practitioners in pediatric anesthesia are mainly attending physicians and other junior clinicians, the generalizability of the study findings to real-world practice may be limited. Future research should address this limitation by including a more representative sample of clinical providers.

## Data Availability

The original contributions presented in the study are included in the article/Supplementary Material, further inquiries can be directed to the corresponding author.
