# Supplementary material for: Current clinical status of perioperative comfort therapy in pediatric anesthesia in China: a national cross-sectional survey
Source: Front Pediatr. 2026 Jun 22;14:1843447. doi: 10.3389/fped.2026.1843447 (PMC13333714; doi:10.3389/fped.2026.1843447)
Supplement: Supplementary file 1 [file Datasheet1.pdf]

Supplementary Material 1

Questionnaire on Pediatric Perioperative Comfort Therapy

(For Pediatric Anesthesiologists)

Dear Colleague,

To optimize pediatric perioperative comfort management and standardize clinical workflows across institutions, we are conducting this nationwide survey. Please answer based on your actual clinical practice. Your participation is anonymous and data will be used for statistical analysis only. Thank you for your support!

SECTION I: BASIC INFORMATION

No.	Question
1	What is the name of the hospital where you work?
2	What is your professional title?
3	How many years have you been engaged in pediatric anesthesia?
4	What is the grade of your hospital?

SECTION II: PREOPERATIVE MANAGEMENT

No.	Question
5	Types of surgery for preoperative visits? *(Multiple selections allowed)*
6	For elective surgery, what is the timing and method of the preoperative visit?
7	What are the qualifications of the personnel conducting the preoperative visit? *(Multiple selections allowed)*
8	Is preoperative anesthesia education provided during the visit?
9	Regarding pre-anesthetic education content, please rank the top 1–4 items in order of importance (1 = most important):
10	What is your preference for preoperative medication? *(Multiple selections allowed)*

11	What non-pharmacological interventions are commonly used in the preoperative waiting area? *(Multiple selections allowed)*
12	Where is anesthesia induction performed?
13	What is the commonly used method of anesthesia induction? *(Multiple selections allowed)*
14	What drugs are commonly used for intravenous induction? *(Multiple selections allowed)*
15	Are parents allowed to accompany the child during anesthesia induction?
16	If parents are present during induction, what are your concerns? *(Multiple selections allowed)*

SECTION III: POSTOPERATIVE MANAGEMENT

No.	Question
17	Are scales used to assess emergence delirium?
18	What is the approximate incidence of emergence delirium in your hospital?
19	What is the first-line measure for managing postoperative agitation?
20	What methods are commonly used to prevent postoperative agitation? *(Multiple selections allowed)*
21	Are parents allowed to accompany the child during the recovery phase (PACU)?
22	Under what circumstances are parents allowed into the PACU for recovery presence? *(Multiple selections allowed)*

SECTION IV: HUMANISTIC CARE

No.	Question
23	What is the impact of parental presence on medical workflow?
24	Do you have any innovative experiences in pediatric perioperative comfort management?
25	What do you consider to be the most urgent clinical pain points in current pediatric perioperative comfort management?

Thank you for your participation!
